# Abnormal Glucose Metabolism in Hispanic Parents of Children with Acanthosis Nigricans

**DOI:** 10.5402/2011/481371

**Published:** 2011-12-25

**Authors:** Ximena Urrutia-Rojas, Walter McConathy, Benjamin Willis, John Menchaca, Mary Luna-Hollen, Khiya Marshall, Andras Lacko, Craig Spellman

**Affiliations:** ^1^Department of Management, Policy, and Community Health, School of Public Health, The University of Texas Health Science Center at Houston, Houston, TX 77030, USA; ^2^Department of Internal Medicine, Texas College of Osteopathic Medicine, University of North Texas Health Science Center, Fort Worth, TX 76107, USA; ^3^Center for Diabetes and Metabolic Disorders, Department of Internal Medicine, Texas Tech University Health Sciences Center, Permian Basin Campus, 701 W 5th Street, Odessa, TX 79763, USA; ^4^Cook Children's Physician Network, Fort Worth, TX 76104, USA; ^5^Department of Behavioral and Community Health, School of Public Health, University of North Texas Health Science Center, Fort Worth, TX 76107, USA; ^6^Department of Molecular Biology and Immunology, Graduate School of Biomedical Sciences, University of North Texas Health Science Center, Fort Worth, TX 76107, USA

## Abstract

*Objective*. Assess the prevalence of abnormal glucose metabolism among Hispanic parents of children with acanthosis nigricans (AN). *Methods*. Hispanic families (*n* = 258) were evaluated for metabolic and anthropometric parameters including fasting glucose levels and AN status. *Results*. Mothers with AN+ children had IFG (17.3%) and 4% had glucose levels ≥126 mg/dL (*P* = 0.028) compared to 7.1% and 1.8% of mothers with AN− children, respectively. Mothers of AN+ children also had greater odds of having impaired fasting glucose levels (OR: 3.917, 95% CI: 1.475–10.404; *P* < 0.004) but this was not the case for fathers (OR: 1.125, 95% CI: 0.489–2.586; *P* = 0.781). Mothers of AN+ children were also more likely to be AN+ (OR: 5.76, 95% CI: 2.98–11.13, *P* < 0.001). Screening discovered glucose levels >126 mg/dL in 9% of fathers with AN+ children. *Conclusions*. Hispanic mothers of AN+ children are at higher risk of carbohydrate metabolism abnormalities. AN in children can be a marker for prevention and delay programs aimed at identifying adults at risk for diabetes.

## 1. Introduction

The emerging epidemic of obesity has been accompanied by an increase in the prevalence of T2D in adolescents and adults. With increasing evidence that childhood obesity precedes insulin resistance and abnormal glucose metabolism and that the risk of diabetes has a strong genetic component [1–3], there is a need to apply screening strategies that identify children, their siblings, and parents at increased risk for T2D and CVD [4].

Random screening is not effective for identifying people at risk for T2D. However, simple indicators, such as acanthosis nigricans (AN), have been recommended by American Diabetes Association (ADA) as one of the markers to identify those at risk for T2D [5]. Acanthosis nigricans, thickening and hyperpigmentation of the skin usually on the neck and intertriginous areas, is frequently associated with obesity, hyperinsulinemia [6, 7], and subsequent risk of T2D [8, 9]. In a study of AN prevalence, 7.1% of unselected 1,412 children were AN positive [10]. The condition was present in 0.5% of white non-Hispanics, (*n* = 440), 5.5% of Hispanics (*n* = 343), and 13.3% of blacks (*n* = 601). In a more recent report, nearly 40% of Native American teenagers aged 10 to 19 years had acanthosis nigricans [6]. Between males and females, AN was equally distributed and more common in obese children. Acanthosis nigricans has also been reported to be familial in some cases [2] and is well known to be associated with hyperandrogenism, polycystic ovary syndrome (PCOS), and cancer [11–13].

Because diabetes can be prevented or delayed [14, 15], it is imperative to identify those individuals at risk. This is highlighted by data showing that as many as 70% of pre-diabetic people will ultimately develop diabetes. The process begins with metabolic dysfunction of which AN is a marker [2, 7]. Over three to five years, approximately 25% of pre-diabetic patients are likely to develop diabetes [16] which carries a high risk of developing heart disease and stroke [17]. Further, identification of undiagnosed diabetes is important because interventions to control fuel metabolism can prevent or delay microvascular complications [18, 19].

Since many adults are reluctant or unable to fully participate in health care, they may be unaware of developing problems or deny the possibility that they are at risk. An avenue to reach these persons may well be through identification of risk factors in their children. In the present study, overweight/obese, AN-positive Mexican-American children were selected as the basis for examining their parents for abnormal glucose metabolism. Our hypothesis was that the presence of AN in children would be associated with abnormal fasting glucose metabolism in their parents. Our findings demonstrate that the mothers of AN-positive children were much more likely to be at risk for T2D than mothers of children without AN. This observation suggests that identification of children that present with AN provides additional opportunities for the detection and prevention of T2D and CVD in Hispanic families. 

## 2. Materials and Methods

### 2.1. Study Groups

In the present study, overweight/obese, AN-positive Mexican-American children were selected as the basis for examining their parents for abnormal glucose metabolism. They were identified as at risk to develop type 2 diabetes at school screenings or referred by primary care providers to a community-based family-focused preventive program that aimed to change eating and physical activity practices among these children and their families. Data from 258 Hispanic families with overweight children were evaluated for the relationship between the presence of AN in children and the presence of glucose and lipid metabolism abnormalities in their parents. Families with at least one child at risk for T2D were invited to participate in the study. ADA guidelines were used to identify AN-positive and AN-negative children [5]. Children had a mean age of 11.1 years, and approximately three fourths (72.3%) were US born. Overall, adults were obese (BMI 30–32) and had a mean age of 36.9 years; most of them were foreign born. Both children (*n* = 446) and parents (*n* = 343, 229 mothers and 114 fathers) were assessed for risk factors for diabetes and CVD. Abnormal glucose is defined as any glucose ≥100 mg/dL or prior diagnosis of T2D. The abnormal glucose group was further broken down into the familiar subsets of impaired fasting glucose (IFG 100–125 mg/dL), glucose ≥126 mg/dL, and known diabetes.

### 2.2. Anthropometric Measurements and Physical Exam

Weight (lbs) and height (in) were converted to metric units to calculate body mass index (BMI, kg/m**^2^**). Waist circumference (cm) was measured with the participant in a reclining position with the tape at the umbilicus and measuring the circumference at the end of the participant's exhalation. Hip circumference was measured at the largest circumference over the buttocks. Average blood pressure (BP) was obtained from two measurements on the right arm. Acanthosis nigricans (AN) was assessed by examination of the neck [6, 7].

### 2.3. Laboratory Studies

All serum constituents were measured by methods and procedures of Quest Diagnostics and included triglycerides (TG), total cholesterol (TC), high-density lipoprotein cholesterol (HDL-C), low-density lipoprotein cholesterol (LDL-C), glucose, insulin, and homocysteine. C-reactive protein (CRP) and leptin were determined using immunoassay kits (Biomerica, Newport, CA, USA, and Biovendor, Brno, Czech Republic). Insulin resistance (homeostasis assessment model for insulin resistance (HOMA-IR)) was calculated as [glucose in mmol/L × insulin in *μ* units/22.5].

### 2.4. Statistical Analyses

All hypothesis tests were two-tailed, and statistical significance was assessed at the 0.05 level using SPSS software. Student's *t*-test was used to compare means, and if data deviated significantly from normality, log-trans-formations or nonparametric methods (the Mann-Whitney U test) were used. Arithmetic means were reported for clarity. Chi-square was used to determine odds ratios and confidence intervals.

### 2.5. Human Subjects Investigation

Approval by the UNTHSC and Cook Children's Hospital IRB's was obtained prior to study.

## 3. Results

The distribution of parents with normal glucose, impaired fasting glucose, glucose ≥126 mg/dL, or known T2D was mapped against the child's AN status (Table 1). Division by gender indicated that differences were significant among mothers (*P* < 0.028) while the fathers displayed no significant difference (*P* < 0.531) although screening indicated undiagnosed diabetes in 9% of the fathers with AN-positive children. As illustrated (Table 2), a mother with an AN-positive child had 5.759 greater odds of being AN-positive and 3.917 greater odds of having impaired fasting glucose. Over two-thirds (*n* = 123, 71.5%) of mothers of AN-positive children were AN positive (Table 3) but only 30.4% (*n* = 17) of mothers of AN-negative children were AN positive (*P* < 0.001). Findings for fathers of AN-positive children followed the same trend but were not significant at *P* < 0.05.

To explore additional differences between mothers of AN-positive and AN-negative children, anthropomorphic and serum parameters were compared (Table 4). Diastolic blood pressure (DBP) was significantly higher in mothers of AN+ children (*P* < 0.037) and BMI showed borderline significance (*P* = 0.060). Waist and waist/hip ratio (W/H) were also higher in mothers of AN-positive children. Evaluation of metabolic parameters showed higher levels of glucose (*P* < 0.036), TG (*P* < 0.035), CRP (*P* < 0.010), and leptin (*P* < 0.098) and lower HDL-C (*P* < 0.029) in mothers of AN-positive children. In contrast, fathers of AN-positive and AN-negative children showed fewer significant differences (Table 5) though their BMI and waist and waist/hip ratio (W/H) were higher among fathers of AN-positive children (*P* < 0.083) as was CRP (*P* < 0.056). This unexpected finding is likely due to the relatively small sample size. A commonality between mothers and fathers of AN-positive children was low HDL-C (*P* < 0.035).

## 4. Discussion

Mexican-American elementary school children with and without AN were selected to determine if their parents had increased risk for diabetes and CVD. The study sought to explore the hypothesis that AN could be a useful marker for undiagnosed T2D or metabolic abnormalities among parents of overweight children with AN. The findings suggest that mothers of AN-positive children are likely to have abnormalities of fuel metabolism compared to mothers of AN-negative children. With respect to fathers, the number of participants was perhaps too low to power this analysis. However, a marker differing the parents, based on offspring AN status, was the observation that the fathers of AN-positive children were more likely to have blood glucose levels ≥126 mg/dL (Table 1). The study suggests that screening children for AN is an effective strategy for identifying adults with pre-diabetes. We suggest that including children in screening programs will be beneficial for improving the quality of diabetes prevention and delay initiatives, by identifying at-risk persons.

A number of studies have pointed to clustering of chronic diseases in families [2, 3, 20, 21]. Williams et al. [22] using only family histories of CHD obtained from a questionnaire distributed to high school students focused on a subset comprising 14% of the general population. Surprisingly, within that group, 72% of all persons with early CHD (men < 55 years, women < 65 years) and 48% with CHD at all ages were identified. This demonstrated that focused screening programs can lead to identification of a substantial percentage (>50%) of the general population at risk for CVD. Our studies suggest that the identification of AN-positive children can serve as a sentinel marker to identify nuclear families at increased risk for T2D and consequently for CVD.

Study limitations relate to the relative small sample size and local nature of the study. Based on a sample size of 229 mothers and 114 fathers, our study had approximately 80% power to detect an odds ratio of 3.4 or greater for abnormal glucose among mothers with AN+ children and 3.2 or greater among fathers. Nonsignificant estimates could have reached significance with a larger sample size. However, our study supports the hypothesis that the presence of AN in a child is associated with abnormal glucose metabolism in their mothers.

## 5. Conclusion

In addition to supporting the hypothesis that the presence of AN in a child is associated with abnormal glucose metabolism in their mothers, our work extends [23] and validates another approach to developing focused screening programs. Specifically, we showed that a physical marker of risk present in children, AN, can be used to identify adult relatives with or at risk for diabetes. Many adults are reluctant or unable to fully participate in health care. They may be unaware of developing problems or deny the possibility that they are at risk. An avenue to reach these persons may well be through identification of risk factors in their children. We suggest using the AN-positive status of offspring as selection criteria to screen families and recommend interventions for prevention or management of diabetes.

##  Ethical Approval

Approval for Human Subjects Investigation by the UNTHSC and Cook Children's Hospital Institutional Review Board (IRB) for the protection of human subjects was obtained prior to the study.

## Figures and Tables

**Table 1:** Frequencies and percentages of parents with impaired glucose or previous diagnosis of diabetes by AN-positive (AN+) and AN-negative (AN−) children.

	Child's AN status	Normal glucose <100* *n* (%)	Impaired fasting glucose 100–125 *n* (%)	Glucose ≥126 *n* (%)	T2D *n* (%)	*P* value*	Total abnormal *n* (%)
Mothers *n* = 229	AN–	51 (91.1%)	4 (7.1%)	1 (1.8%)	0 (0%)	0.028	5 (8.9%)
	AN +	125 (72.3%)	30 (17.3%)	7 (4.0%)	11 (6.4%)		48 (27.7%)
Fathers *n* = 114	AN–	25 (67.6%)	11 (29.7%)	1 (2.7%)	0 (0%)	0.531	12 (32.4%)
	AN +	50 (64.9%)	19 (24.7%)	7 (9.1%)	1 (1.3%)		27 (35.1%)

*Chi-square.

**Table 2:** Odds ratio for abnormal glucose and acanthosis nigricans in mothers and fathers with AN-positive and AN-negative children.

Parent	Odds ratio (95% CI)	*P* value
Abnormal glucose		
Mothers	3.917 (1.475, 10.404)	0.004*
Fathers	1.125 (0.489, 2.586)	0.781*
Acanthosis nigricans		
Mothers	5.759 (2.980, 11.129)	0.001*
Fathers	1.971 (0.886, 4.386)	0.094*

*Chi-square.

**Table 3:** Frequencies and percentages of mothers and fathers with acanthosis nigricans by AN-positive and AN-negative children.

Child's AN status		Parent AN−	Parent AN+
	Mothers		
Child AN–		39 (69.6%)	17 (30.4%)
Child AN+		49 (28.5%)	123 (71.5%)**
	Fathers		
Child AN–		22 (59.5%)	15 (40.5%)
Child AN+		32 (42.7%)	43 (57.3%)***

Chi-square. **P < 0.0001; ***P < 0.111.

**Table 4:** Anthropomorphic and serum parameters of mothers with AN-negative and AN-positive children.

	AN negative child (*n* = 56)		AN positive child (*n* = 173)		
	Mean	SE	Mean	SE	P*
Anthropomorphic parameters					
Age	37.4	1.0	35.9	0.4	
Weight (kg)	75.6	2.4	80.5	1.4	0.091
Height (m)	1.57	0.01	1.57	0.01	
BMI	30.5	0.9	32.6	0.5	0.060
Waist (cm)	93.3	1.8	96.9	1.2	
Hip size (cm)	109.8	1.7	112.4	1.1	
Waist hip ratio	0.85	0.01	0.86	0.01	
Blood Pressure					
Systolic BP	115.3	2.3	119.1	1.4	
Diastolic BP	67.8	1.4	71.0	0.7	0.037
Serum Parameters					
Carbohydrate					
Glucose	92.3	3.4	96.7	2.0	0.036
Insulin	8.5	0.8	9.1	0.5	
HOMA	2.00	0.20	2.28	0.14	
Lipid					
TG	118.7	7.2	139.8	5.2	0.035
TC	184.6	4.3	182.0	2.4	
LDL-C	109.6	3.7	106.6	2.1	
HDL-C	51.2	1.6	47.4	0.8	0.029
TC/HDL-C	3.8	0.1	4.0	0.1	
LDL-C/HDL-C	2.3	0.1	2.4	0.1	
Other					
Homocysteine	5.2	0.2	5.7	0.1	0.010
CRP (mg/L)	4.9	0.9	5.1	0.5	
Leptin (mg/L)	28.4	2.4	33.5	1.6	0.098

* Student's *t*-test. All *P* values at *P*≤ 0.10 listed.

**Table 5:** Anthropomorphic and serum parameters of fathers with AN-negative and AN-positive children.

	AN-negative child (*n* = 37)		AN-positive child (*n* = 77)		
	Mean	SE	Mean	SE	P*
Anthropomorphic parameters					
Age	39.9	0.9	38.6	0.7	
Weight (kg)	87.6	2.4	90.2	1.7	
Height (m)	1.70	0.01	1.69	0.01	
BMI	30.3	0.6	31.4	0.5	
Waist (cm)	98.7	1.5	100.5	1.2	
Hip size (cm)	105.3	1.1	104.8	0.9	
Waist hip ratio	0.94	0.01	0.96	0.01	0.083
Blood pressure					
Systolic BP	124.6	2.0	127.7	2.2	
Diastolic BP	74.9	1.4	76.0	1.5	
Serum parameters					
Carbohydrate					
Glucose	99.8	3.8	110.7	5.4	
Insulin	8.2	1.0	9.2	0.9	
HOMA	2.01	0.25	2.54	0.25	
Lipid					
TG	198.4	22.9	203.2	18.4	
TC	209.9	9.0	202.3	4.8	
LDL-C	126.3	8.1	121.6	3.9	
HDL-C	43.9	1.8	40.1	0.9	0.035
TC/HDL-C	5.1	0.3	5.2	0.2	
LDL-C/HDL-C	3.0	0.2	3.1	0.1	
Other					
Homocysteine	7.6	0.4	7.9	0.4	
CRP (mg/L)	1.8	0.2	2.8	0.4	0.056
Leptin (mg/L)	11.3	1.5	10.0	0.9	

*Student's *t*-test. All *P* values at *P*≤ 0.10 listed.
